# The Effectiveness of a Smartphone Application for Tinnitus Relief

**DOI:** 10.3390/healthcare11172368

**Published:** 2023-08-22

**Authors:** Hye Yoon Seol, Mini Jo, Il Joon Moon

**Affiliations:** 1 Medical Research Institute, Sungkyunkwan University School of Medicine, Suwon 16419, Republic of Korea; 2 Hearing Research Laboratory, Samsung Medical Center, Seoul 06351, Republic of Korea; 3 Department of Otolaryngology-Head & Neck Surgery, Samsung Medical Center, Sungkyunkwan University School of Medicine, Seoul 06351, Republic of Korea

**Keywords:** tinnitus, smartphone application, audiology

## Abstract

Background: There has been a growing interest in the provision of smartphone- or internet-based tinnitus management. Studies have shown a positive impact of the smartphone applications on tinnitus symptoms. However, research into a relatively long-term effect of these applications on tinnitus relief as well as sound preferences has been sparse. This study explored the potential benefit of a tinnitus application in tinnitus relief over a period of six months. Methods: Twenty-two participants with subjective tinnitus were enrolled in the study. Puretone audiometry, tinnitus pitch and loudness matching, stress assessment, and questionnaires were completed at the initial visit and three and six months after the first visit. The participants used the tinnitus application for six months. Results: A significant reduction in subjective tinnitus loudness and annoyance and subjective stress level was observed. The Tinnitus Handicap Inventory scores were also significantly decreased after six months. The participants also reported high satisfaction with the application. Music and environmental sounds were the most preferred sound stimuli. Conclusions: The findings of this study demonstrate the potential benefit of the tinnitus application for tinnitus improvement.

## 1. Introduction

Tinnitus refers to sounds that individuals hear without the presence of external sounds [1]. Tinnitus can be classified into subjective and objective tinnitus. Subjective tinnitus is a sound perception that is only audible to the affected individuals. In contrast, objective tinnitus refers to sound that originates within the body and is audible to both the affected individual and others. Tinnitus can occur due to various causes such as hearing loss and auditory nerve pathologies, among others, but there are also cases where the exact cause remains unclear [2]. Individuals typically present with various characteristics (i.e., severity, frequency, sounds, onset). For some, tinnitus could be something fairly easy to manage with music or noise (i.e., sound generators). However, some people experience tinnitus that significantly reduces their quality of life; there are studies showing that, along with decreased communication efficiency and academic or job performance, individuals could also be suicidal [3,4]. In addition to the worsening quality of life and suicide, there is a significant correlation between tinnitus severity and psychiatric symptoms. Molnar et al. (2022) investigated the correlation between depression, anxiety, and tinnitus handicap in 102 individuals with tinnitus. A correlation between tinnitus and both depression and anxiety was observed, with a stronger correlation between tinnitus and depression [5]. With its high prevalence and negative impact on various aspects of life, tinnitus needs to be managed appropriately based on individuals’ tinnitus characteristics. Most cases of tinnitus cannot be definitively treated, only symptomatic therapy is possible in most cases. Instead, healthcare professionals try various approaches for tinnitus relief. Examples include pharmaceutics, hearing technology, cognitive behavioral therapy, and magnetic stimulation [6,7,8,9,10,11,12]. Hearing technology, such as hearing aids and sound generators, could be beneficial especially for those with hearing impairment. In a study conducted by Surr et al. (1985), 200 new hearing aid users completed a questionnaire regarding tinnitus and the effect of hearing aids on tinnitus. Almost half of the new hearing aid users reported that their tinnitus was relieved after the use of the devices [9]. Folmer and Carroll (2006) examined the long-term effectiveness of ear-level devices including hearing aids and sound generators for tinnitus. A total of 150 participants were recruited and divided into three groups: hearing aid, sound generator, and no device. Individuals in the no device group were recommended to try other types of sound therapy, such as relaxation CDs. Six to forty-eight months after the initial visit, follow-up questionnaires were mailed to the participants to explore the effectiveness of the devices in tinnitus relief. The results revealed that the use of the ear-level devices (hearing aids or sound generators) significantly improved tinnitus [11].

In the era of the fourth industrial evaluation and digital therapeutics, there has been increasing research interest in smartphone- or internet-based tinnitus management [13,14,15,16,17]. Numerous tinnitus applications have been created and they range from those that utilize notched music or band-pass noise for tinnitus management to those that offer relaxation exercises, attentional focus exercises, and diary-writing features [12,18]. These types of interventions are advantageous when it comes to rehabilitation cost, location, portability, and self-administration which could ultimately enhance patient compliance as well [19,20,21], so studies are currently underway to assess their effectiveness. Mehdi et al. (2020) conducted a systematic review on smartphone applications for tinnitus. There are numerous applications available for individuals experiencing tinnitus, enabling them to engage in tinnitus management through the utilization of smartphone-based cognitive behavioral therapy, sound therapy, questionnaires, and more. Through the use of these applications, individuals can gain insights into the various characteristics of tinnitus. This empowers them to readily identify the specific factors or situations that affect their perception towards tinnitus. More specifically, for tinnitus sound therapy, Kutyba et al. (2022) utilized the ReSound Tinnitus Relief application to investigate its impact on tinnitus improvement. Fifty-two tinnitus patients used the application at least for 30 min per day for six months and a significant improvement in tinnitus was observed when assessed by the Tinnitus Handicap Inventory (THI) and Tinnitus Functional Index [15]. In a pilot study conducted by Engelke et al. (2023), they developed an application for tinnitus sound therapy and provided counseling to the participants for 12 weeks. Significant improvement in tinnitus was observed at the end of the 12-week period [16]. Although pre-existing studies have shown a positive impact of smartphone-based tinnitus management, the scientific evidence as to the effectiveness of the smartphone application in tinnitus relief in the long term and the participants’ perspectives towards the smartphone-based tinnitus management is underwhelming [13,14]. Thus, this study explored the feasibility of a smartphone application for tinnitus relief in individuals with tinnitus.

## 2. Material and Methods

### 2.1. Participants

A total of 22 individuals who were diagnosed with tinnitus at Samsung Medical Center participated in the study. The inclusion criteria were as follows: (1) age 19 years or older, (2) subjective and chronic tinnitus (≥3 months), (3) no history of a neurological, cognitive, learning, or language disorder, (4) native Korean speakers, and (5) use an Android smartphone. Individuals who did not meet the inclusion criteria were excluded from the study. All experimental procedures were approved by Samsung Medical Center’s Institutional Review Board and an informed consent document was obtained from the participants.

### 2.2. Procedures

The study consisted of a total of three visits. After informed consent was obtained, the initial visit (visit 1) began with hearing, tinnitus, and stress assessments, and the installation of the tinnitus application. The participants were informed about how to use the application during their participation while the application was being installed on their mobile phone. A guide for the tinnitus application was provided to the participants. They also completed three questionnaires at the initial visit: THI, Beck Depression Index (BDI), and a general questionnaire about tinnitus characteristics and subjective stress level. After three months, the participants made their second visits (visit 2) and completed the same tests and a questionnaire about sound management through the tinnitus application. The average time intervals of the second visit were five days. During the second visit, participants were asked to provide responses regarding whether listening to music through the tinnitus application for three months had helped in alleviating their tinnitus, which type of music was the most effective for tinnitus relief, and their subjective stress level. The final visit (visit 3) was made six months after the initial visit with the same tests. Similar to the second visit, during the third visit, participants were asked to evaluate the effect of the use of the tinnitus application in tinnitus relief and subjective stress level. An additional questionnaire evaluating the tinnitus application was administered.

### 2.3. Hearing and Tinnitus Assessment

Otoscopy was performed to examine the outer ear canal and the tympanic membrane. Pure-tone thresholds from 250 Hz to 8000 Hz for both ears were obtained in a sound booth using an AudioStar Pro (Grason-Stadler, Eden Prairie, MN, USA) audiometer and insert earphones. Tinnitus frequency and loudness matching were performed using the same equipment. Tinnitus frequency matching was performed using a two-alternative forced-choice method; the participants were presented with two tones and chose the one that was the most similar to the frequency of the sound that they hear. Once the tinnitus frequency was identified, tinnitus loudness matching began with the same frequency tone. The tone was presented at a level below the participants’ thresholds first and gradually increased in a 1 dB step size until the loudness of the tone presented was reported to be the same as the participants’ tinnitus.

### 2.4. Stress Assessment

Subjective stress level was assessed using VAS on a scale of 0 (not stressed at all) to 10 (extremely stressed). The participants evaluated their subjective stress levels during each visit.

### 2.5. Tinnitus Relief through a Smartphone Application

**Figure 1:**
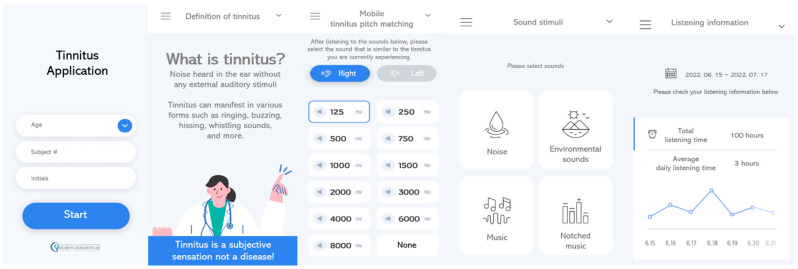
A screenshot of the tinnitus application.

The tinnitus application had five sections: study information, tinnitus education, mobile tinnitus pitch and loudness matching, sound management, and listening information. Figure 1 shows images of the tinnitus education as well as sound management sections. The procedures regarding the research study were provided in study information. Tinnitus education provided information about tinnitus in general; participants had a chance to read about the definition, prevalence, current methods to manage tinnitus, and tips for tinnitus relief in this page. For instance, individuals were informed that there are various ways (i.e., listening to music, hearing devices, etc.) to relieve tinnitus since the majority of tinnitus cases cannot be definitively treated. Mobile tinnitus pitch and loudness matching was available in the tinnitus application for the participants to be aware of their tinnitus characteristics as well as to create notched music as sound stimuli. There were pure-tone stimuli ranging from 125 to 8000 Hz, and participants performed a task of finding the sound that most closely resembled their own tinnitus by pressing each pure-tone stimulus and listening to it. The participants completed mobile tinnitus pitch and loudness matching with an experienced audiologist at the initial visit. Sound management provided four categories for various sound stimuli: noise, environmental sound, music, and notched music. The noise category had white, brown, and pink noise. White noise has equal energy across all audible frequencies, while brown noise has more lower-frequency energy. Pink noise, on the other hand, has equal energy per octave. The environmental sound category had environmental sounds, such as crickets, campfire, ocean waves, raindrops, etc. Various musical genres, such as classical music, children’s songs, country music, and jazz, were available in the music category. Notched music was created with a 1-octave notch width for all songs in the music category. The participants did not have any restrictions on the type, number, volume, and listening time of the sound stimuli they listened to. After listening to the sounds, they responded to two survey items. The first item assessed the degree of tinnitus relief experienced after listening to the sounds, while the second item asked about the sound that provided the most relief or was most preferred for tinnitus relief. In the second item, participants were presented with the four sound categories mentioned earlier and an option for “none”. If they found any of the sounds effective for tinnitus relief, they selected one of the four categories. If they did not have a preferred sound or did not find any sound effective for tinnitus relief, they chose “none”. Lastly, on the listening information page, participants had access to information regarding the types of sounds they listened to, the duration of listening, the degree of tinnitus relief, and their preferred sound stimulus for tinnitus relief. Information regarding the degree of tinnitus relief and preferred sounds was based on the responses to the two survey items conducted after listening to the sounds. During the entire process, the experienced audiologist first provided guidance on how to use the application, demonstrating to patients how to download and listen to the sound stimuli using a demo smartphone. After the demo, participants were given the opportunity to navigate through the desired menu on the demo smartphone, choose some sounds they would like to listen to, listen to the sounds, and check the listening information. This served as a check to ensure that participants were able to use the application effectively on their own.

### 2.6. Questionnaire

To evaluate the effectiveness of the tinnitus application in tinnitus relief before and after three and six months of application use, the Korean version of the THI and BDI were administered [22,23,24,25]. The Korean version of the THI consists of a total of 25 items, organized into functional subscale, emotional subscale, and catastrophic subscale. Each item is rated on a scale of “no”, “sometimes”, and “yes”, with scores of 0, 2, and 4 points, respectively. Based on the total score which is calculated by summing up the points from all three subscales, tinnitus severity can be categorized into no handicap (0 to 16 points), mild (18 to 36 points), moderate (38 to 56 points), severe (58 to 76 points), and catastrophic (78 to 100 points). The Korean version of the BDI consists of 21 items and is designed to assess symptom severity on a scale of 0 to 3 points. The potential scores range from 0 to 63 and can be categorized into not depressed (0 to 9), mildly depressed (10 to 15), moderately depressed (16 to 23), and severely depressed (24 to 63).

Individuals’ tinnitus characteristics were also investigated using a tinnitus sound management questionnaire developed by the authors previously [26]. Here, the participants indicated the onset and duration of their tinnitus, subjective loudness and annoyance, previous methods they tried to alleviate tinnitus (i.e., supplements, listening to music), and the effectiveness of the attempted methods for tinnitus relief. Information about subjective loudness and annoyance were obtained at visits 1, 2, and 3. Information about sound preferences was obtained through questionnaires at visits 2 and 3. Subjective loudness and annoyance were measured using VAS. An additional questionnaire evaluating the tinnitus application on the 5-point Likert scale (ranging from “strongly disagree” to “strongly agree”) was administered at visit 3. This questionnaire was divided into four main categories: usability, efficiency, design, and satisfaction. Under the usability category, participants were asked to evaluate whether the application was easy to use and if the information provided in the application was clearly explained. The efficiency category focused on assessing whether it was easy to access the desired menus within the application (i.e., tinnitus education to listening information) and if the application functioned reliably. In the design category, participants evaluated the overall design consistency and the appropriateness of text and icon sizes. Lastly, under the satisfaction category, participants evaluated their overall satisfaction with using the application. The participants also provided responses regarding their willingness to recommend this application to others and other sound stimuli that they would like to listen to.

### 2.7. Statistical Analysis

Statistical analysis was performed using SigmaPlot 15.0. Subjective stress level and the scores of the Korean version of BDI and THI were analyzed using the one-way RM ANOVA as it passed the normality test (Shapiro–Wilk). A *p*-value of 0.05 was considered significant.

## 3. Results

### 3.1. Participant Characteristics

Out of the 22 participants, 5 were males and 17 were females. The age range of the participants was from 24 to 71 years old with a mean age of 52.2 years (SD = 13.4). The four-frequency (500, 1000, 2000, and 4000 Hz) averages of pure-tone thresholds were 24.4 (SD = 15.2) dB in the right ear and 26.9 (SD = 17.2) dB in the left ear, corresponding to normal to mild hearing loss.

### 3.2. Tinnitus Characteristics

Table 1 describes the tinnitus characteristics of the participants in this study. The number of ears with tinnitus was 20 for the right ear and 16 for the left ear. The mean duration of tinnitus was 75.1 months (SD = 71.1). Based on the results of tinnitus pitch matching through audiometry, tinnitus frequency was divided into three categories: low (125–750 Hz), mid (1000–3000 Hz), and high (4000–8000 Hz). High frequency was the most common tinnitus frequency followed by low- and mid-frequencies. The mean tinnitus loudness was 35.7 dB (SD = 21.6). VAS (0-not loud/annoyed at all, 5-neutral, 10-extremely loud/annoyed) was used to assess subjective loudness and annoyance of tinnitus. The mean VAS for subjective loudness was 4.9 (SD = 1.9) while the mean VAS for subjective annoyance was 5.4 (SD = 2.6). Out of the 22 participants, 10 had never received any treatment related to tinnitus, while 12 had received some form of treatment. Among these 12 individuals, they reported having tried supplements, such as Ginkgo biloba and Aronamin C+, using a sound generator, or listening to music. However, these methods did not provide any significant relief for tinnitus; when evaluating the effectiveness of the treatments on a scale of 0 (no effect) to 10 (significant effect) using the VAS, both supplements, music, and sound generators showed results ranging from 1 to 3.

### 3.3. Stress

The mean VAS for subjective stress levels were 5.8 (SD = 2.4), 4.7 (SD = 1.9), and 4.0 (SD = 2.4) at visits 1, 2, and 3, respectively. There was a statistical significance between the visits (*p* < 0.001). The Bonferroni correction method was applied for all *p*-values to correct for multiple comparisons (*p* < 0.016). There was a statistical difference between the subjective stress levels at visit 1 and visit 2 (*p* = 0.014) and between visit 1 and 3 (*p* < 0.001). These results indicate that individuals felt less stressed about their tinnitus after listening to various sound stimuli for three and six months.

### 3.4. Questionnaires

BDI and THI scores were analyzed using the one-way RM ANOVA. Table 2 illustrates the results for BDI and THI. The mean BDI scores were 8.0 (SD = 4.8), 6.4 (SD = 4.1), and 5.7 (SD = 5.4) at visits 1, 2, and 3. Although the mean BDI scores decreased over time, there was no statistical significance between the visits (*p* = 0.123). The THI scores, on the other hand, significantly decreased over the six-month period (*p* = 0.024); when the Bonferroni correction method was applied for all *p*-values, statistical significance was observed for THI scores between visits 1 and 3 (*p* = 0.006). This indicates that after six months of listening to the sound stimuli, participants’ perceived tinnitus handicap severity significantly decreased.

Changes in subjective loudness and annoyance were analyzed using the same statistical method (Table 3). Statistical significance was observed for both subjective loudness (*p* = 0.041) and annoyance (*p* < 0.001). For subjective loudness, when the Bonferroni correction method was applied for all *p*-values, statistical significance was observed between visits 1 and 3 (*p* = 0.014); the participants felt that the loudness of their tinnitus significantly decreased after six months. The same correction method was applied for all *p*-values for subjective annoyance as well. There was a statistical significance between visits 1 and 2 (*p* = 0.015), between visits 1 and 3 (*p* < 0.001), and between visit 2s and 3 (*p* = 0.002). These results suggest a significant reduction in participants’ subjective annoyance after listening to the sound stimuli for three and six months.

### 3.5. Sound Preference

Sound preferences were examined at visits 2 and 3 and are described in Table 4. At visit 2, music was preferred the most (50%), followed by environmental sound (36.4%), notched music (9.1%), and noise (4.5%). However, at visit 3, the participants responded that they preferred environmental sounds the most (45.5%). Approximately 31.8% of them preferred music, while 18.2% and 4.5% of participants preferred notched music and noise, respectively. The sound preferences for the unilateral and bilateral tinnitus groups were also compared. Regardless of the laterality of tinnitus, music was preferred the most at visit 2 and environmental sound was preferred the most at visit 3. When making comparisons based on the tinnitus frequency range (low, mid, and high), for those with low tinnitus frequency, similar to aforementioned results, music and environmental sounds were the most preferred sound stimuli at visits 2 and 3, respectively. However, those who have tinnitus frequency in the mid-frequency range, environmental sound was the most preferred sound category at visit 2 and environmental sound and notched music were both preferred the most at visit 3. Lastly, for those with a high tinnitus frequency, music was preferred the most at visit 2, but music and environmental sounds were the most preferred sound stimuli at visit 3. Some additional sound stimuli that participants hoped to be included as options for tinnitus relief were pure tones, animal sounds, trot music, and baby crying sounds.

### 3.6. Tinnitus Application Evaluation

The results of the tinnitus application evaluation are presented in Table 5. The application was reported to be easy to use and was running stably. Moreover, the participants were highly satisfied with the design and the use of the application. On the 5-point Likert scale (1—strongly disagree, 3—neutral, 5—strongly agree), the average scores for the ease of application, application stability, application design, and overall satisfaction were 4.4, 4.1, 3.9, and 3.9, respectively. A total of 64% of the participants reported on the questionnaire that they would like to recommend the tinnitus application to others.

## 4. Discussion

This study explored the potential benefit of a smartphone application in tinnitus relief. After using the smartphone application for six months, the participants not only experienced a significant reduction in their tinnitus and subjective stress levels, but were also highly satisfied with the application use which is, according to previous literature, an important factor when individuals choose applications they use [27]. The THI scores, subjective loudness, as well as annoyance scores significantly decreased after using the tinnitus application for six months. These findings are consistent with previous literature demonstrating the positive impact of the smartphone application on tinnitus [15,17,28]. As stress and depression have been reported to be correlated with tinnitus [5,29,30], subjective stress levels were measured and the Korean version of the BDI was performed at each visit. The results showed that subjective stress levels reduced significantly after three and six months. The tinnitus application evaluation questionnaire revealed a high level of satisfaction and willingness to recommend the tinnitus application to others. In addition, the type of sound stimuli that could possibly be the most effective for tinnitus was explored in this study. Nagaraj and Prabhu (2020) conducted a systematic review on internet- and smartphone-based applications for tinnitus and mentioned that most applications focus on the provision of various sounds instead of incorporation of other components, such as education and counselling [12]. The application that the authors developed for the purpose of this study not only provided a variety of sound stimuli for listening but also offered education and listening information. This way, participants were able to learn basic information about tinnitus (definition, methods that can be utilized for tinnitus relief, etc.) before selecting and listening to sound stimuli. After listening to the sound stimuli, they could access the “listening information” menu to view the sound stimuli they listened to the most, their preferred sound stimuli, and information about the degree of improvement in tinnitus. This provided an advantage of not only listening to the sound stimuli but also gaining awareness of their tinnitus characteristics and assessing the degree of improvement in tinnitus relief associated with listening to the sound stimuli.

This study is meaningful in that there was a relatively long-term effect of the tinnitus application in tinnitus improvement; in addition, overall device performance, satisfaction, and sound preferences were investigated based on tinnitus characteristics. In general, the participants preferred music and environmental sounds the most. When divided into tinnitus frequency range, notched music was also one of the most preferred sounds for those who have their tinnitus frequency in the mid-frequency range. It is worth nothing that these types of intervention methods need to be informative, but also easy to be utilized to increase adherence to interventions [21]. Given the results of the study, smartphone-based interventions for tinnitus could possibly bring forth positive changes in the management of tinnitus. Subsequent studies with larger sample sizes and various participant characteristics and more longitudinal studies are necessary as certain aspects of this type of tinnitus intervention, such as sound preference, might change depending on sample size and tinnitus characteristics (i.e., duration of tinnitus).

In terms of limitations, this application was only available for Android smartphone users and needs to be translated into the official language of the country. It is also important to note that a detailed tinnitus diagnosis and management by a specialist is necessary before using this type of application. Searchfield et al. (2017) highlighted important aspects of tinnitus relief research and methods utilizing sound stimuli. They mentioned the ambiguity in the effectiveness of sound-based interventions in tinnitus therapy, which is attributed to various mechanisms of sound that can influence tinnitus, such as energetic masking, pattern recognition, attention, and cognition [18]. Mckenna and Irwin (2008) also argued that the mechanisms attributed to tinnitus sound therapy may not align with the actual effects, suggesting that the benefits could stem from cognitive or psychological factors rather than purely auditory ones [31]. Currently, there is a lack of established research paradigms that consider various aspects of sound management for tinnitus relief. In addition, there is a lack of heterogeneity in tinnitus applications—there are limited sound management options that can genuinely be regarded as personalized, thus failing to fully utilize their proposed mechanisms [18,32]. As a result, there is also a scarcity of guidelines for selecting and implementing appropriate sounds for rehabilitation strategies based on the diverse characteristics of tinnitus. In order to conduct research on tinnitus relief using sound stimuli and evaluate its effect comprehensively, there is a need to establish a paradigm that takes into account multiple factors that can contribute to tinnitus relief [33,34]. Overall, the findings from our study could provide baseline data for some effective sounds based on tinnitus characteristics, but ample work is still needed to confirm the feasibility of the application and the most effective type of sound for different tinnitus characteristics. As mentioned earlier, internet- and smartphone-based interventions offer benefits in terms of cost-effectiveness, flexibility in location, portability, and the ability for individuals to self-administer them. For example, individuals living in rural areas may find it difficult to commute to clinics or hospitals for tinnitus treatment. In such cases, using a tinnitus application after a first visit and basic diagnostics allows them to conveniently listen to sound stimuli and attempt tinnitus relief from the comfort of their own homes. This way, they could actively participate in tinnitus management without the need to visit the hospital every time for treatment. These types of interventions could improve patient compliance, but it is also important to keep in mind that smartphone-based interventions require individuals to own and be able to use the smartphones which might not be possible for certain patients [35]. Bommakanti et al. (2020) interviewed 150 participants about smartphone ownership, sociodemographics, and tuberculosis treatment perceptions towards the directly observed therapy (conventional method) and video-observed therapy. The study reported that those who are older, male, less educated, or had a lower annual income might not have access to mobile health interventions due to the possibility of not having smartphones [35]. While conducting tinnitus sound therapy research, considering various aspects is important; it seems that there are still many factors to consider when developing tinnitus applications based on these research findings and implementing them in clinical settings. Therefore, careful considerations should be made when employing these types of interventions in clinical practice.

## 5. Conclusions

The findings of the study demonstrate the potential benefit of the tinnitus application in decreasing tinnitus symptoms. The use of smartphone applications, after a specialist’s diagnosis, could provide advantages in terms of cost-effectiveness, location flexibility, portability, and the convenience for individuals to self-administer them. Further research with larger participant groups and diverse characteristics, along with longitudinal studies, is essential to explore certain aspects of this tinnitus intervention, such as sound preference, which may vary based on sample size and tinnitus characteristics.

## Figures and Tables

**Table 1:** Tinnitus characteristics at baseline (visit 1).

Laterality	8 (Unilateral), 14 (Bilateral)
Duration (months)	75.1 (SD = 71.1)
Onset	17 (sudden), 5 (gradual)
Frequency	7 (low), 6 (mid), 9 (high)
Loudness	35.7 dB (SD = 21.6)
Sound	2 (buzzing), 13 (pure tone), 5 (noise), 2 (other)
Consistency	11 (consistent), 11 (fluctuating)
Subjective loudness	4.9 (SD = 1.9)
Subjective annoyance	5.4 (SD = 2.6)

**Table 2:** BDI and THI results over six months.

Questionnaire	Mean Scores (SD)			*p*
	Visit 1	Visit 2	Visit 3	
BDI	8.0 (4.8)	6.4 (4.1)	5.7 (5.4)	0.123
THI	33.2 (20.8)	29.4 (18.0)	24.8 (19.3)	0.024 *

BDI = the Korean version of the Beck Depression Index, THI = the Korean version of the Tinnitus Handicap Inventory; * *p* < 0.005.

**Table 3:** Subjective loudness and annoyance results over six months.

Variable	Mean Scores (SD)			*p*
	Visit 1	Visit 2	Visit 3	
Loudness	4.9 (1.9)	4.4 (2.1)	4.0 (2.1)	0.041 *
Annoyance	5.4 (2.6)	4.6 (2.8)	3.6 (2.4)	<0.001 **

* *p* < 0.005, ** *p* < 0.001.

**Table 4:** Sound preference over six months.

Visit	Preference Ranking			
	1	2	3	4
2	Music	Environmental sound	Notched music	Noise
3	Environmental sound	Music	Notched music	Noise

**Table 5:** Evaluation of the tinnitus application.

Domain	Question	Average Score
Accessibility	Was the application easy to use?	4.5
	Was it easy to understand the information in the application?	4.2
Efficiency	Was it easy to switch between menus?	4.1
	Was the application running stably?	4.2
Design	Was the design of the application appropriate?	3.9
	Were the text size and color appropriate?	4.0
	Was the icon size appropriate?	4.0
Satisfaction	Overall, were you satisfied with the application?	4.1

## Data Availability

The data supporting the findings of this study are available from the corresponding author, I.J.M., upon reasonable request. The data were not publicly available because of ethical considerations.
